# Evaluation of Serum Insulin-Like Growth Factor 1 (IGF-1) Among Controlled and Uncontrolled Type 2 Diabetes Mellitus in a Geriatric Population From North India

**DOI:** 10.7759/cureus.96716

**Published:** 2025-11-12

**Authors:** Arjun Dev J, Sartaj Hussain, Manish Kumar Singh, Mohd Arif, K K Sawlani, Kauser Usman, Sanjay Khattri

**Affiliations:** 1 Pharmacology, Autonomous State Medical College, Gonda, Gonda, IND; 2 Pharmacology, All India Institute of Medical Sciences Vijaypur, Jammu, Jammu, IND; 3 Biochemistry, Government Medical College, Badaun, Sirsauli, IND; 4 Pharmacology, Autonomous State Medical College Amethi, Tiloi, IND; 5 Internal Medicine, King George's Medical University, Lucknow, IND; 6 Pharmacology and Therapeutics, King George's Medical University, Lucknow, IND

**Keywords:** biomarker, geriatric patients, glycemic control, insulin-like growth factor-1 (igf-1), type 2 diabetes mellitus (t2dm)

## Abstract

Introduction: Insulin-like growth factor-1 (IGF-1) plays an important role in glucose and lipid metabolism, yet its relationship with glycemic status in elderly type 2 diabetes mellitus (T2DM) remains incompletely defined. This study aimed to assess serum IGF-1 concentrations in geriatric patients from North India with controlled and uncontrolled T2DM.

Materials and methods: An age- and sex-matched case-control design was used, enrolling 100 participants: 50 with controlled T2DM (HbA1c <7%) and 50 with uncontrolled T2DM (HbA1c >7%). Clinical and biochemical assessments included fasting plasma glucose, HbA1c, lipid profile, fasting insulin, insulin resistance indices (homeostasis model assessment of insulin resistance (HOMA-IR), homeostasis model assessment of β-cell function (HOMA-B), tumor necrosis factor-alpha (TNF-α), and serum IGF-1 concentrations.

Results: Serum IGF-1 was significantly reduced in uncontrolled T2DM compared with controlled patients (4.16 vs. 7.70 ng/mL, p <0.001). IGF-1 demonstrated negative correlations with age, duration of diabetes, fasting glucose, HbA1c, total cholesterol, triglycerides, low-density lipoprotein cholesterol (LDL-C), fasting insulin, HOMA-IR, and TNF-α. On multivariate regression, advancing age and HbA1c independently predicted IGF-1 levels. Receiver operating characteristic* (*ROC) curve analysis identified a cut-off of 7.32 ng/mL for IGF-1, yielding 92% sensitivity and 60% specificity in predicting uncontrolled diabetes.

Conclusion: Serum IGF-1 is significantly lower in uncontrolled geriatric T2DM and correlates with markers of glycemic control, insulin resistance, and inflammation. It may represent a promising biomarker for monitoring glycemic status in older adults with T2DM.

## Introduction

Type 2 diabetes mellitus (T2DM) is a metabolic disorder characterized by hyperglycemia, insulin resistance, and inflammation [1]. As the global population ages, the prevalence of T2DM is rising among the geriatric population, leading to increased morbidity and mortality worldwide [2]. Effective glycemic control is crucial for preventing acute and chronic complications in the geriatric population; however, achieving this remains a significant challenge in clinical practice [3].

The insulin/insulin-like growth factor 1 (IGF-1) signaling (IIS) axis plays a central role in growth, development, and metabolic regulation [4]. IGF-1 is a structural homolog of insulin that regulates glucose homeostasis and insulin sensitivity [5]. Disruption of the IIS axis and altered levels of IGF-1 are seen in metabolic disorders like diabetes and metabolic syndrome [6,7]. Various studies investigated the association between IGF-1 levels and T2DM, but IGF-1 levels in controlled versus uncontrolled T2DM in the geriatric population remain unexplored.

Investigating and understanding the association between serum IGF-1 levels and glycemic control will improve patient outcomes and facilitate the development of targeted interventions. The aim of this study was to investigate serum IGF-1 levels in controlled and uncontrolled T2DM within the geriatric population and evaluate its potential as a biomarker for metabolic control in this vulnerable group. We aim to determine whether serum IGF-1 levels are significantly altered in controlled versus uncontrolled geriatric T2DM patients and to explore how glycemic control affects IGF-1 levels.

## Materials and methods

This was an age- and sex-matched prospective case-control study conducted in the Department of Pharmacology, in collaboration with the Departments of Medicine and Pathology, at King George's Medical University, Lucknow, Uttar Pradesh, India, a tertiary care teaching and research institute in North India. Written informed consent was obtained from all subjects, and the study was approved by the Institutional Ethics Committee, King George's Medical University (Ref. code: XVI-PGTSC-IIA/P78). All procedures in the study were conducted in accordance with the Declaration of Helsinki.

Study population

A total of 100 patients with T2DM were recruited from the outpatient clinic, 50 with controlled T2DM and 50 with uncontrolled T2DM. Participants were unrelated individuals with similar ethnicity from Lucknow and other adjoining cities of Northern India. The diagnosis of T2DM was made as per International Diabetes Federation (IDF) criteria, 2017 [8], and then patients were screened for eligibility.

The inclusion criteria encompassed age ≥60 years and the use of oral hypoglycemic agents for ≥6 months, while the exclusion criteria comprised type 1 diabetes, cardiovascular diseases, cerebrovascular diseases, liver diseases, autoimmune diseases, inflammatory diseases, bacterial and viral infections, genetic disorders, patients on insulin therapy, and the use of steroids.

Subjects with T2DM were classified into two groups: a control group consisting of patients with controlled T2DM (HbA1c < 7) and a case group comprising patients with uncontrolled T2DM (HbA1c ≥ 7), using an HbA1c cutoff value of 7 to define control status [9].

Clinical and biochemical measurements

After overnight fasting, five ml of venous blood was drawn from the antecubital vein by a standard venipuncture method and divided into three parts: 1.5 ml in a fluoride vial for fasting glucose estimation, 1.5 ml in a K3 ethylenediaminetetraacetic acid (EDTA) vial for HbA1c and 2 ml in a plain vial to facilitate serum separation for lipid profile and enzyme-linked immunosorbent assay (ELISA). Fasting plasma glucose (FPG), lipid profile, HbA1c, serum insulin levels, and lipid profile were estimated on the same day of collection. The remaining serum was stored at -20 °C for ELISA.

FPG, total cholesterol (TC), triglycerides (TG), and high-density lipoprotein cholesterol (HDL-C) levels in serum samples were estimated by auto-analyzer (Selectra pro-XL; ELITechGroup B.V., Puteaux, France) and relevant kits. The quantification of HbA1c was achieved through the D-10 TM high-performance liquid chromatography (HPLC) (Bio-Rad Laboratories, Inc., Hercules, California, United States) and its kit. Low-density lipoprotein cholesterol (LDL-C) levels were determined by applying the Friedewald equation. Serum insulin level was quantified by the Alinity-I analyzer (Abbott Laboratories, Abbott Park, Illinois, United States) and the related kit. The serum IGF-1 and TNF-α levels were measured using the sandwich ELISA method using the commercially available Lablisa® kit (LABReCON, New Delhi, India), according to the manufacturer’s protocol. HOMA-IR and HOMA-B were computed based on the values of fasting insulin and FPG, utilizing the standard formula.

Statistical analysis

The normality of the distribution of data was checked by using the Shapiro-Wilk test. The data is presented as median (interquartile range (IQR)) or mean (standard deviation) based on normality or number (percentage). Mann-Whitney U test and chi-square test were used to compare continuous or categorical data, respectively. Data were log-transformed before performing univariate and multivariate regression analysis. Multivariate linear regression analysis with a forward selection method was applied to determine the independent predictors of the IGF-1 levels. The variables with a P value ≤ 0.10 in the univariate regression analysis were used in multivariate linear regression. Binary logistic regression analysis was applied to calculate the odds of uncontrolled diabetes for IGF-1. The receiver operating characteristic curve (ROC) was used to investigate the diagnostic ability of IGF-1. The optimal cut-off value of IGF-1 was determined by the Youden index. The ROC curve analysis was performed using MedCalc Statistical Software version 23.3.7 (MedCalc Software, Ostend, Belgium). The rest of the statistical analyses were performed in IBM SPSS Statistics for Windows, version 25.0 (IBM Corp., Armonk, New York, United States). Two-sided P < 0.05 was considered statistically significant.

## Results

The study included 50 patients with controlled T2DM as controls and 50 patients with uncontrolled T2DM as cases. The demographic, clinical, and laboratory parameters of the case and control groups are displayed in Table 1. The mean age and the gender distribution were similar across both groups. The median duration of diabetes was significantly higher in the uncontrolled group (13 years; IQR: 9-17.25) compared to the controlled group (10 years; IQR: 5-15) (P=0.023). Body mass index (BMI) did not differ significantly between groups. Systolic blood pressure (SBP) and diastolic blood pressure (DBP) were comparable between the groups. FPG and HbA1c levels were markedly elevated in the uncontrolled group compared to the controlled group (FPG: 163.5 mg/dL vs. 108.5 mg/dL, P<0.001; HbA1c: 8.05% vs. 6.6%, P<0.001). Among lipid profiles, TC, TG, HDL-C, and very-low-density lipoprotein cholesterol (VLDL-C) showed no significant differences. While low-density lipoprotein cholesterol (LDL-C) levels were higher in the uncontrolled group (86.6 mg/dL; IQR: 67.99-112.36) compared to the controlled group (74.86 mg/dL; IQR: 66.55-91.33) (P=0.042). Markers of insulin resistance and beta-cell function demonstrated significant differences. The median fasting insulin was significantly higher in the uncontrolled group (12.6 μIU/mL; IQR: 6.2-23.05) compared to the controlled group (10.7 μIU/mL; IQR: 7.2-14.2). However, this difference was statistically non-significant (P=0.340). The HOMA-IR was significantly higher in the uncontrolled group (4.72; IQR: 2.31-9.69) compared to the controlled group (2.81; IQR: 1.9-3.79) (P=0.001). Conversely, the HOMA-B was significantly lower in the uncontrolled group (43.89; IQR: 25.4-78.56) compared to the controlled group (84.7; IQR: 69.04-116.66) (P<0.001). TNF-α levels were elevated in the uncontrolled group (29.41 pg/mL; IQR: 22.08-38.88) compared to the controlled group (18.55 pg/mL; IQR: 15.8-21.41) (P<0.001). IGF-1) levels were significantly reduced in the uncontrolled group (4.16 ng/mL; IQR: 2.65-6.49) compared to the controlled group (7.7 ng/mL; IQR: 5.95-9.52) (P<0.001).

**Table 1:** Comparison of anthropometric, clinical, and lab parameters of controlled and uncontrolled type 2 diabetes mellitus subjects. * indicates the effect size for the chi-square test (Cramér’s V); the remaining values represent the effect size in terms of Cohen’s d. The Mann-Whitney U test or chi-square test was used for comparing data. Cohen’s d was used to calculate the effect size for the Mann–Whitney U test. BMI, body mass index; DBP, diastolic blood pressure; FPG, fasting plasma glucose; HbA1c, glycated hemoglobin; HDL-C, high-density lipoprotein cholesterol; HOMA-IR, homeostasis model assessment of insulin resistance; HOMA-B, homeostasis model assessment of β-cell function; IGF-1, insulin-like growth factor 1; LDL-C, low-density lipoprotein cholesterol; SBP, systolic blood pressure; SD, TC, total cholesterol; TNF-α, tumor necrosis factor-alpha; VLDL-C, Very-low-density lipoprotein cholesterol; IQR, interquartile range

Parameter	Control Diabetes (n=50), median (IQR)	Uncontrolled Diabetes (n=50), median (IQR)	P-value	Effect size/degree of freedom
Age (years)	65 (62-68)	63 (62-68)	0.261	0.225
Gender (Male/Female)	23 (46.0%)/27 (54.0%)	25 (50.0%)/25 (50.0%)	0.689	0.04^*^
Duration of DM (years)	10 (5-15)	13 (9-17.25)	0.023	0.467
BMI (kg/m^2^)	23.23(21.96-26.15)	23.53 (21.94-25.55)	0.934	0.017
SBP (mmHg)	140 (136-148)	140 (134-150)	0.438	0.155
DBP (mmHg)	80 (78-87.25)	81 (78-90)	0.382	0.174
FPG (mg/dl)	108.5 (98.75-116.5)	163.5 (142-201.25)	<0.001	2.891
HbA1c (%)	6.6 (6.2-6.8)	8.05 (7.4-8.83)	<0.001	3.397
TC (mg/dl)	160.6 (139.18-179.03)	163.1 (148.73-203.53)	0.072	0.366
TG mg/dl)	111.65 (96.75-154.25)	128.3 (103.85-195.35)	0.053	0.395
HDL-C (mg/dl)	56.2 (48.45-66.95)	53.54 (42.48-65.15)	0.258	0.228
LDL-C (mg/dl)	74.86 (66.55-91.33)	86.6 (67.99-112.36)	0.042	0.415
VLDL-C (mg/dl)	23.05 (19.15-30.85)	24.68 (19.86-33.87)	0.432	0.158
Fasting insulin (μIU/mL)	10.7 (7.2-14.2)	12.6 (6.2-23.05)	0.340	0.192
HOMA-IR	2.81 (1.9-3.79)	4.72 (2.31-9.69)	0.001	0.701
HOMA-B	84.7 (69.04-116.66)	43.89 (25.4-78.56)	<0.001	1.224
TNF-α(pg/ml)	18.55 (15.8-21.41)	29.41 (22.08-38.88)	<0.001	1.680
IGF-1 (ng/ml)	7.7 (5.95-9.52)	4.16 (2.65-6.49)	<0.001	1.357

Univariate linear regression analysis was conducted to determine the relationship between serum IGF-1 levels and various variables. The IGF-1 and other variables were log_e_ transformed before conducting the univariate regression analysis. The results of univariate linear regression are displayed in Table 2. IGF-1 showed a significant inverse correlation with age, duration of T2DM, SBP, DBP, FPG, HbA1c, TC, TG, LDL-C, VLDL-C, fasting insulin, HOMA-IR, and TNF-α (P < 0.05). To determine the independent predictors of serum IGF-1 levels, we did multivariate linear regression with the forward selection method with an entry criterion of p-value ≤ 0.10 for the variables in univariate linear regression analysis.

**Table 2:** Univariate linear regression analysis for Loge IGF1 BMI, body mass index; DBP, diastolic blood pressure; FPG, fasting plasma glucose; HbA1c, glycated hemoglobin; HDL-C, high-density lipoprotein cholesterol; HOMA-IR, homeostasis model assessment of insulin resistance; HOMA-B, homeostasis model assessment of β-cell function; IGF-1, insulin-like growth factor 1; LDL-C, low-density lipoprotein cholesterol; SBP, systolic blood pressure; SD, TC, total cholesterol; TNF- α, tumor necrosis factor-alpha; VLDL-C: Very-low-density lipoprotein cholesterol.

Parameter	β	t	P-value
Log_e_ Age (years)	-0.21	-2.14	0.035
Log_e_ BMI (kg/m^2^)	0.002	-0.02	0.983
Log_e_ duration of DM (years)	-0.21	-1.59	0.036
Log_e_ SBP (mmHg)	-0.29	-2.99	0.003
Log_e_ DBP (mmHg)	-0.33	-3.42	0.001
Log_e_ FPG (mg/dl)	-0.63	-8.07	<0.001
Log_e_ HbA1c (%)	-0.68	-9.20	<0.001
Log_e_ TC (mg/dl)	-0.58	-7.10	<0.001
Log_e_ TG (mg/dl)	-0.21	-2.09	0.039
Log_e_ HDL-C (mg/dl)	-0.16	-1.57	0.119
Log_e_ LDL-C (mg/dl)	-0.38	-4.06	<0.001
Log_e_ VLDL-C (mg/dl)	-0.20	-2.01	0.048
Log_e_ fasting insulin (μIU/mL)	-0.37	-3.89	<0.001
Log_e_ HOMA-IR	-0.54	-6.37	<0.001
Log_e_ HOMA-B	0.17	1.75	0.084
Log_e_ TNF-α (pg/ml)	-0.48	-5.37	<0.001

The results of multivariate linear regression analysis are shown in Table 3. Age (β = -0.71, P <0.001) and HbA1c (β = -0.028, P <0.001) were independent predictors of serum IGF-1 levels. Binary logistic regression analysis revealed that the occurrence of uncontrolled T2DM decreased with increasing concentration of IGF-1 with an odds ratio (OR) of 0.60 (95%CI = 0.49-0.75, P < 0.001) (Table 4).

**Table 3:** Multivariate linear regression analysis for the independent predictor of Loge IGF1 HbA1c, glycated hemoglobin. Forward selection method Input variables: p ≤0.10 in univariate linear regression analysis.

Parameter	β	t	P-value
Log_e_ HbA1c (%)	-0.71	-10.21	<0.001
Log_e_ Age (years)	-0.28	-4.00	<0.001

**Table 4:** Binary logistic regression analysis of IGF1 for the odds of uncontrolled diabetes IGF-1, insulin-like growth factor 1.

Parameter	B	Wald	OR (95% CI)	P
IGF-1 (ng/ml)	0.51	21.44	0.60 (0.49-0.75)	<0.001

**Figure 1:**
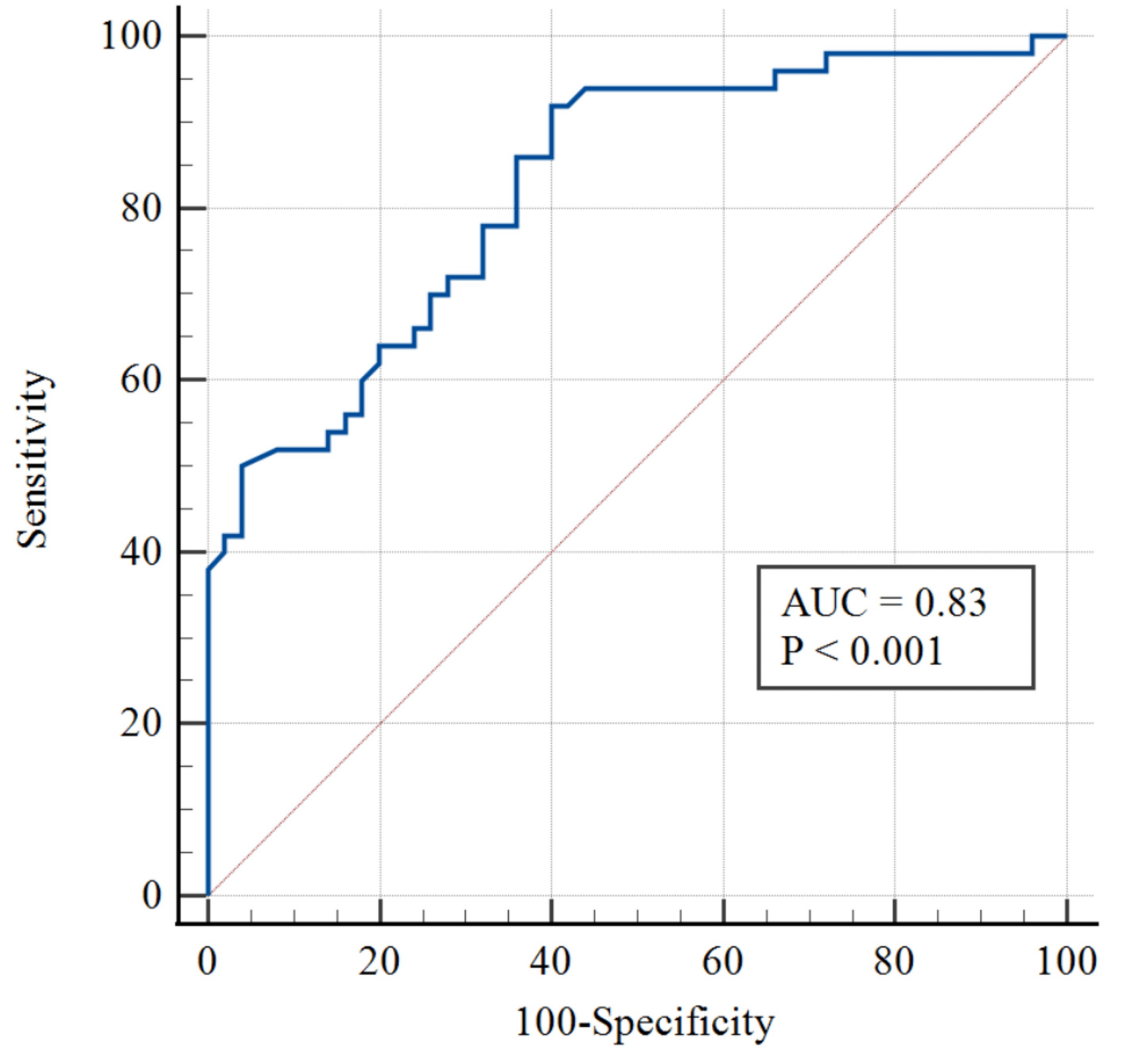
ROC curve illustrating the diagnostic utility of IGF-1 in predicting uncontrolled type 2 diabetes mellitus in the geriatric population. IGF-1, insulin-like growth factor 1, ROC, receiver operating characteristic; AUC: area under the curve

The diagnostic utility of IGF-1 for discriminating uncontrolled T2DM from controlled T2DM was determined by ROC analysis, and the results are shown in Table 5. The area under the curve (AUC) for IGF-1 was 0.83 (95% CI, 0.74 to 0.89) with P < 0.001. We applied the Youden index (YI) to determine the optimal cut-off value of IGF-1. The optimal cut-off value for IGF-1 was ≤7.32 ng/mL, which can differentiate uncontrolled T2DM from controlled T2DM with a sensitivity of 92.00% and a specificity of 60.00% (Figure 1).

**Table 5:** Cut-off points and diagnostic utility of serum IGF-1 level for uncontrolled diabetes IGF-1, insulin-like growth factor 1.

Parameter	Optimal cut-off value	AUC (95% CI)	P-value	Sensitivity (95% CI)	Specificity (95% CI)
IGF-1 (ng/ml)	≤7.32	0.83 (0.74-0.89)	<0.001	92.00 (80.8-97.8)	60.00 (45.2-73.6)

## Discussion

Our study shows that older adults with uncontrolled T2DM have significantly lower serum IGF-1 compared to well-controlled peers. IGF-1 was inversely associated with age, glycemic markers (fasting glucose, HbA1c), insulin resistance (HOMA-IR), TNF-α, and atherogenic lipids, while higher levels were linked to better insulin sensitivity. Elevated IGF-1 was also tied to lower odds of uncontrolled diabetes, suggesting a possible protective role. To the best of our knowledge, this is among the first investigations in a geriatric Indian cohort to highlight IGF-1 as a potential biomarker of glycemic control, offering fresh insights into the interaction between aging, diabetes, and the insulin-IGF axis. Age-related declines in circulating IGF-1 further contextualize these observations [10].

Mechanistically, IGF-1 shares structural similarity with proinsulin but signals through distinct receptors [11]. Acting via the IGF-1 receptor and PI3K-AKT pathway, it regulates cellular glucose metabolism [12]. Produced mainly in the liver, IGF-1 secretion is driven by growth hormone and portal insulin [13]. Beyond insulin’s primary role in glucose homeostasis, IGF-1 enhances peripheral glucose uptake and suppresses hepatic gluconeogenesis [14]. Circulating IGF-1 exists largely bound to IGFBPs, particularly IGFBP-3, which modulate its bioavailability and may independently influence glucose metabolism [15]

Our findings are broadly consistent with several prior studies, though some discrepancies exist across different populations. Suda et al. (2016) observed markedly lower IGF-1 in Japanese patients with very poorly controlled T2DM (HbA1c ≥12%) compared to those with HbA1c <12%, and noted that improvements in HbA1c during follow-up were paralleled by rises in IGF-1 [16]. Similarly, a study by Sesti et al. (2005) showed significantly lower IGF-1 in T2DM subjects as compared to healthy controls [17]. Prospective data also support this pattern: Sandhu et al. (2002) found that individuals with IGF-1 ≥152 µg/L had nearly 50% lower risk of impaired glucose tolerance or diabetes compared to those below this threshold [18]. Other large-scale studies highlight non-linear associations. In Danish adults (n=3,354), IGF-1 displayed a U-shaped relationship with insulin resistance, with both low and high levels linked to elevated HOMA-IR [19]. Similarly, pooled analyses from DETECT (Diabetes and Cardiovascular Risk Evaluation: Targets and Essential Data for Commitment of Treatment) and SHIP (Studying the Hurdles of Insulin Prescription) cohorts (n=7,777) demonstrated that IGF-1 extremes (below 10th or above 90th percentile) increased diabetes risk compared to mid-range concentrations, confirming a U-shaped pattern [20]. Colao et al. (2008) also reported higher IGF-1 in NGT compared to IGT or diabetes, reinforcing its decline with worsening glucose tolerance [21].

Not all studies, however, report reduced IGF-1. A recent Iraqi study found paradoxically higher IGF-1 in poorly controlled T2DM (HbA1c >8%) versus HbA1c <8%, with levels rising alongside HbA1c and disease duration [22]. This discrepancy may reflect population differences: the Iraqi cohort was middle-aged and obese, where hyperinsulinemia may sustain IGF-1 despite poor control. In contrast, our geriatric, leaner Indian cohort likely exhibits impaired insulin secretion (low HOMA-B), limiting hepatic IGF-1 production despite hyperglycemia. Indeed, Suda et al. noted that IGF-1 correlated with fasting C-peptide (endogenous insulin output) but not BMI, and increased with HbA1c improvement [16]. Thus, in older insulin-deficient patients, IGF-1 declines with worsening glycemia, whereas in younger insulin-resistant patients, levels may remain normal or high until later disease stages.

Inflammation link

Chronic inflammation offers another explanation for low IGF-1 in uncontrolled diabetes. We observed a strong inverse correlation between IGF-1 and TNF-α, a key pro-inflammatory cytokine known to drive insulin resistance. Experimental studies have shown that TNF-α exposure markedly suppresses IGF-1 gene expression in target tissues and increases IGF-binding protein levels that sequester IGF-1 [23]. For example, in vascular smooth muscle cells, TNF-α reduced IGF-1 production by ~85% while increasing insulin-like growth factor binding protein-3 (IGFBP-3), thereby lowering bioactive IGF-1 [23]. This mechanism contributes to plaque instability in atherosclerosis and likely mirrors systemic changes in diabetes. Our cohort showed higher TNF-α (29.4 vs 18.5 pg/mL) and lower IGF-1 (4.16 vs 7.70 ng/mL) in uncontrolled versus controlled patients, consistent with this pattern. A meta-analysis further linked elevated TNF-α with reduced IGF-1 as joint risk factors for ischemic stroke [24]. Parallel epidemiologic data indicate that individuals with higher interleukin-6 (IL-6) and lower IGF-1 levels face an elevated risk of metabolic syndrome [25]. Thus, hyperglycemia, insulin resistance, and inflammation synergistically suppress IGF-1 in uncontrolled T2DM.

Lipid metabolism

In the present study, IGF-1 was inversely correlated with TC, TG, LDL-C, and VLDL, suggesting that lower levels contribute to a more atherogenic lipid profile, even though mean lipid values did not differ between groups, likely due to lipid-lowering therapy. Most IGF-1 circulates bound to IGFBP-3, which regulates its bioavailability and exerts IGF-1 independent metabolic effects via nuclear receptors such as RXR-α and PPAR-γ [26]. Experimental studies further show that IGFBP-3 overexpression induces insulin resistance and dyslipidemia, while partial IGF-1 deficiency enhances gluconeogenic and lipogenic gene expression changes reversible with IGF-1 replacement [27]. Clinically, higher IGF-1 has been associated with favorable lipid profiles; for example, Song et al. (2016) reported a positive correlation between IGF-1 and HDL-C in T2DM patients [28]. Taken together, these findings imply that IGF-1 insufficiency contributes not only to hyperglycemia but also to dyslipidemia, compounding cardiovascular risk.

Correlates in our cohort and prior evidence

In our study, IGF-1 showed inverse associations with age, diabetes duration, blood pressure, glycemia, insulin resistance, and inflammation. IGF-1 level normally peaks in early adulthood and declines with age, a trend confirmed in population data [10,29]. Since diabetes mimics accelerated aging, geriatric T2DM patients show further IGF-1 reduction. While one study in hospitalized T2DM patients found no correlation with BMI, diabetes duration, FPG, or HbA1c [16], another in metabolic syndrome patients reported negative associations with glucose, insulin, TG, LDL-C, VLDL-C, and HOMA-IR [30]. Similarly, Friedrich et al. (2012) identified a U-shaped link between IGF-1 and HOMA-IR in Danish adults [19]. 

Protective associations and biomarker potential

In our study, higher IGF-1 levels were linked to lower odds of uncontrolled diabetes in elderly T2DM patients. This is consistent with prior evidence: a prospective study showed that IGF-1 above the median halved the risk of glucose intolerance or diabetes over ~4.5 years [18], and a 7,777-subject European cohort revealed a U-shaped association, where both low and high IGF-1 increased diabetes risk [20]. Together, these findings suggest that moderate IGF-1 levels are protective for glucose regulation.

Diagnostic performance (ROC)

IGF-1 showed strong discriminatory ability for uncontrolled T2DM in the elderly, with an AUC of 0.83 (p < 0.001). A cut-off of ≤7.32 ng/mL yielded 92% sensitivity and 60% specificity. Compared with Colao et al. (2008), who reported 75.9% sensitivity and 85.7% specificity [21]. The differences in specificity between our findings and those of Colao et al. may be attributed to variations in study populations, cutoff values, or methodological approaches. Overall, the results support IGF-1 as a potential biomarker for identifying uncontrolled diabetes in geriatric patients, warranting validation in larger studies.

Limitations

This was a cross-sectional study from a single center, so it cannot prove cause and effect, and the results apply mainly to elderly North Indian patients. The sample size was small, and excluding patients on insulin or with major comorbidities further limited the scope. A non-diabetic control group was not included, reducing the strength of comparisons. IGF-1 was measured only in total form, without considering age-adjusted ranges, free IGF-1, binding proteins, or growth hormone, which restricts mechanistic insights. Therefore, the results should be viewed as preliminary, and larger, long-term studies are needed to confirm them.

## Conclusions

This case-control study demonstrates that lower circulating IGF-1 levels are strongly associated with poor glycemic control in geriatric patients with T2DM, and are linked to greater insulin resistance, longer disease duration, inflammation (elevated TNF-α), and adverse lipid profiles. These findings align with broader evidence identifying IGF-1 insufficiency as a hallmark of metabolic syndrome and dysglycemia. Consequently, IGF-1 may serve as an integrative biomarker of metabolic health in elderly diabetics, reflecting the collective status of insulin signalling, inflammation, and metabolic dysregulation.

Importantly, higher IGF-1 correlates with better metabolic control, suggesting that maintaining adequate IGF-1 could be protective against both hyperglycemia and atherogenic lipid changes. Taken together, the intricate relationship between IGF-1 and T2DM highlighted here warrants further exploration, as confirmation in larger longitudinal studies could open a new dimension in the management of diabetes in the aging global population.
